# Supplementary material for: A robust, low-cost instrument for real-time colorimetric isothermal nucleic acid amplification
Source: PLoS One. 2022 Sep 30;17(9):e0256789. doi: 10.1371/journal.pone.0256789 (PMC9524685; doi:10.1371/journal.pone.0256789)
Supplement: S1 File — (Top) PN dye absorbance spectrum measured at 90.09 μM (78.125 μg/mL) with a 1 cm path length. (Bottom) PN dye absorbance at 590 nm as a function of concentration, normalized to a 1 cm path length, showing a linear regression fit with y(0) = 0. Extinction coefficient can be determined from the slope of this line: ε = 30,289. (DOCX) [file pone.0256789.s001.docx]

**S1. PN Dye Spectroscopy.** (Top) PN dye absorbance spectrum measured at 90.09 μM (78.125 μg/mL) with a 1 cm path length. (Bottom) PN dye absorbance at 590 nm as a function of concentration, normalized to a 1 cm path length, showing a linear regression fit with y(0) = 0. Extinction coefficient can be determined from the slope of this line: ε = 30,289.

**
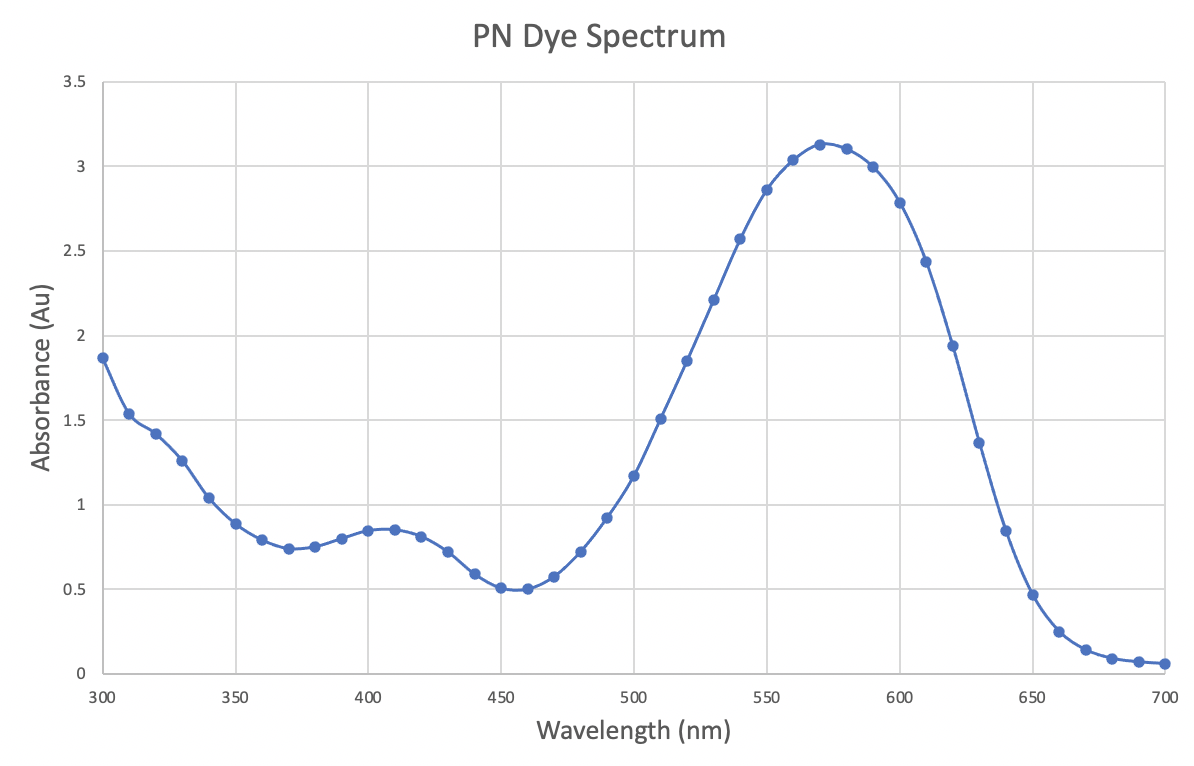
**

**
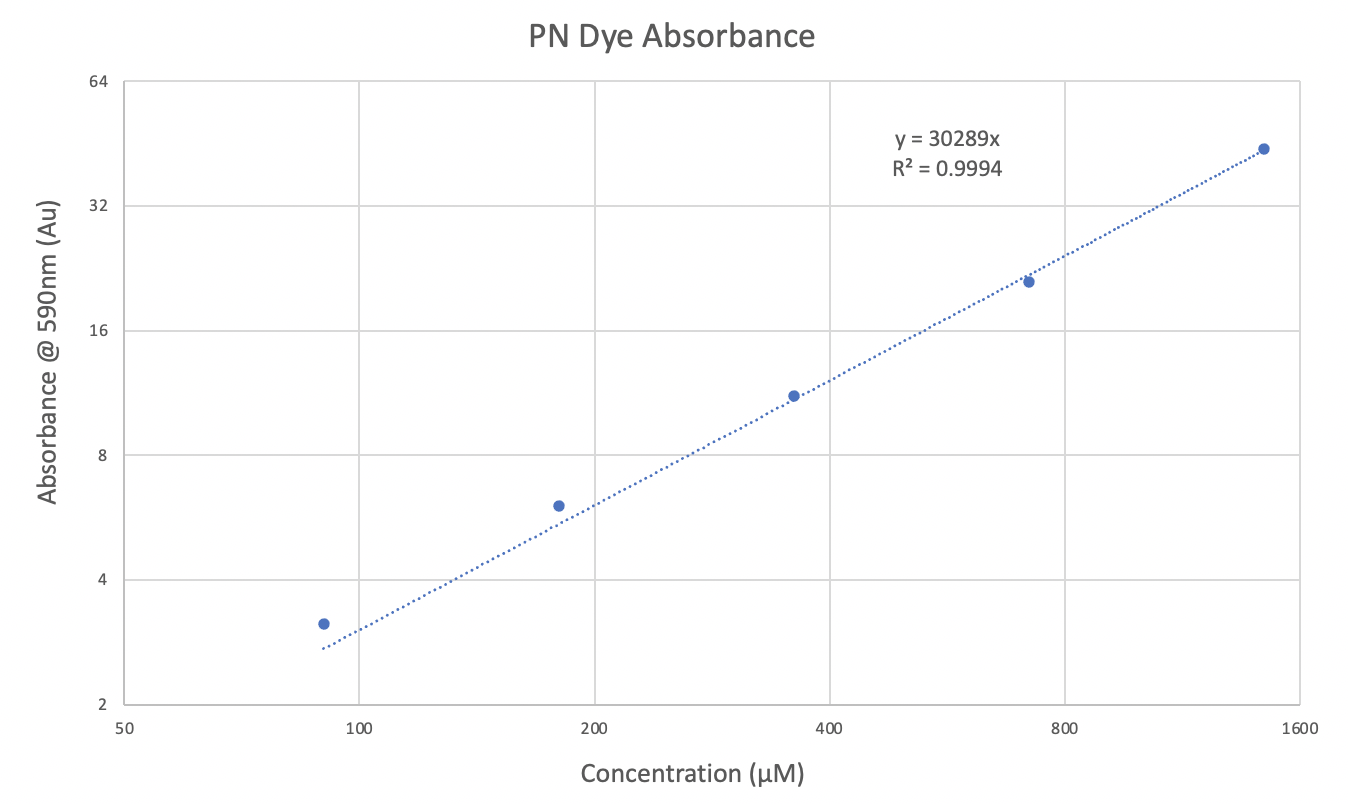
**
