# Supplementary material for: A robust, low-cost instrument for real-time colorimetric isothermal nucleic acid amplification
Source: PLoS One. 2022 Sep 30;17(9):e0256789. doi: 10.1371/journal.pone.0256789 (PMC9524685; doi:10.1371/journal.pone.0256789)
Supplement: S2 File — This comparison examined variability across multiple LARI instruments as well as variability across tubes within a single instrument. (DOCX) [file pone.0256789.s002.docx]

**S2. One-way ANOVA of instrument-response parameters.** This comparison examined variability across multiple LARI instruments as well as variability across tubes within a single instrument.

**Comparison Across LARIs**

Midpoint Concentration

|  | SS | DF | MS | F | p-unc | np2 |
| --- | --- | --- | --- | --- | --- | --- |
| LARI # | 3939.70 | 4 | 984.9258 | 13.014821 | 1.595e-07 | 0.486266 |
| Within Group | 4162.24 | 55 | 75.67725 | N/A | N/A | N/A |

*Statistical differences exist at the p=0.05 level*

Midpoint Slope

|  | SS | DF | MS | F | p-unc | np2 |
| --- | --- | --- | --- | --- | --- | --- |
| LARI # | 98.29540 | 4 | 24.57385 | 4.086742 | 0.005695 | 0.229119 |
| Within Group | 330.7186 | 55 | 6.013066 | N/A | N/A | N/A |

*Statistical differences exist at the p=0.05 level*

Midpoint Signal

|  | SS | DF | MS | F | p-unc | np2 |
| --- | --- | --- | --- | --- | --- | --- |
| LARI # | 581324 | 4 | 145331.2 | 10.49252 | 0.000002 | 0.432815 |
| Within Group | 761801 | 55 | 13850.94 | N/A | N/A | N/A |

*Statistical differences exist at the p=0.05 level*

**Comparison Across Tubes**

Midpoint Concentration

|  | SS | DF | MS | F | p-unc | np2 |
| --- | --- | --- | --- | --- | --- | --- |
| Tube # | 1780.019 | 11 | 161.8199 | 1.228636 | 0.294762 | 0.219702 |
| Within Group | 6321.932 | 48 | 131.7069 | N/A | N/A | N/A |

*No statistical differences at the p=0.05 level*

Midpoint Slope

|  | SS | DF | MS | F | p-unc | np2 |
| --- | --- | --- | --- | --- | --- | --- |
| Tube # | 195.7052 | 11 | 17.79129 | 3.660291 | 0.000826 | 0.456172 |
| Within Group | 233.3100 | 48 | 4.860622 | N/A | N/A | N/A |

*Statistical differences exist at the p=0.05 level*

Midpoint Signal

|  | SS | DF | MS | F | p-unc | np2 |
| --- | --- | --- | --- | --- | --- | --- |
| Tube # | 429642 | 11 | 39058.40 | 2.052365 | 0.043369 | 0.319882 |
| Within Group | 913484 | 48 | 19030.92 | N/A | N/A | N/A |

*Statistical differences exist at the p=0.05 level*
