# Supplementary material for: A robust, low-cost instrument for real-time colorimetric isothermal nucleic acid amplification
Source: PLoS One. 2022 Sep 30;17(9):e0256789. doi: 10.1371/journal.pone.0256789 (PMC9524685; doi:10.1371/journal.pone.0256789)
Supplement: S3 File — No statistical differences were observed in TTR, signal amplitude, or signal slopes across 5 LARI instruments. (DOCX) [file pone.0256789.s003.docx]

**S3. One-way ANOVA of instrument-to-instrument variability in signal analysis results.** No statistical differences were observed in TTR, signal amplitude, or signal slopes across 5 LARI instruments.

TTR

|  | SS | DF | MS | F | p-unc | np2 |
| --- | --- | --- | --- | --- | --- | --- |
| LARI # | 0.840095 | 4 | 0.210024 | 1.070609 | 0.374382 | 0.035902 |
| Within Group | 22.55980 | 115 | 0.196172 | N/A | N/A | N/A |

*No statistical differences at the p=0.05 level*

Starting Signal Amplitude

|  | SS | DF | MS | F | p-unc | np2 |
| --- | --- | --- | --- | --- | --- | --- |
| LARI # | 2.536e+05 | 4 | 63403.09 | 1.713475 | 0.15175 | 0.056247 |
| Within Group | 4.255e+06 | 115 | 37002.63 | N/A | N/A | N/A |

*No statistical differences at the p=0.05 level*

Signal Ratio

|  | SS | DF | MS | F | p-unc | np2 |
| --- | --- | --- | --- | --- | --- | --- |
| LARI # | 3.122562 | 4 | 0.780640 | 1.067258 | 0.376043 | 0.035793 |
| Within Group | 84.11616 | 115 | 0.731445 | N/A | N/A | N/A |

*No statistical differences at the p=0.05 level*

Slope

|  | SS | DF | MS | F | p-unc | np2 |
| --- | --- | --- | --- | --- | --- | --- |
| LARI # | 0.002820 | 4 | 0.000705 | 1.000771 | 0.410223 | 0.033639 |
| Within Group | 0.081012 | 115 | 0.000704 | N/A | N/A | N/A |

*No statistical differences at the p=0.05 level*
