# Supplementary material for: A robust, low-cost instrument for real-time colorimetric isothermal nucleic acid amplification
Source: PLoS One. 2022 Sep 30;17(9):e0256789. doi: 10.1371/journal.pone.0256789 (PMC9524685; doi:10.1371/journal.pone.0256789)
Supplement: S4 File — Minor statistical differences exist along the length of the heating block. (DOCX) [file pone.0256789.s004.docx]

**S4. One-way ANOVA of tube-to-tube variability in signal analysis results.** Minor statistical differences exist in TTR along the length of the heating block.

TTR Comparison

|  | SS | DF | MS | F | p-unc | np2 |
| --- | --- | --- | --- | --- | --- | --- |
| Tube # | 3.902476 | 11 | 0.354771 | 2.8514 | 0.001363 | 0.082679 |
| Within Group | 43.29808 | 348 | 0.124420 | N/A | N/A | N/A |

*Statistical differences exist at the p=0.05 level*

*See next page for pairwise Tukey comparisons between tube locations*

Starting Signal Comparison (Positive Samples)

|  | SS | DF | MS | F | p-unc | np2 |
| --- | --- | --- | --- | --- | --- | --- |
| Tube # | 1.887e+06 | 11 | 171582 | 1.616537 | 0.092235 | 0.048613 |
| Within Group | 3.693e+07 | 348 | 106142 | N/A | N/A | N/A |

*No statistical differences at the p=0.05 level*

Signal Ratio Comparison (Positive Samples)

|  | SS | DF | MS | F | p-unc | np2 |
| --- | --- | --- | --- | --- | --- | --- |
| Tube # | 13.87276 | 11 | 1.261161 | 1.09361 | 0.365263 | 0.033413 |
| Within Group | 401.3167 | 348 | 1.153209 | N/A | N/A | N/A |

*No statistical differences at the p=0.05 level*

Slope Comparison

|  | SS | DF | MS | F | p-unc | np2 |
| --- | --- | --- | --- | --- | --- | --- |
| Tube # | 0.00397 | 11 | 0.003610 | 0.153019 | 0.99302 | 0.004814 |
| Within Group | 0.82073 | 348 | 0.002358 | N/A | N/A | N/A |

*No statistical differences at the p=0.05 level*

Pairwise Tukey Test for Tube-to-Tube Variability of TTR

| A | B | mean(A) | mean(B) | diff | SE | T | p-tukey |
| --- | --- | --- | --- | --- | --- | --- | --- |
| T1 | T10 | 9.643667 | 9.360667 | 0.283 | 0.091075 | 3.10733 | 0.085192 |
| T1 | T11 | 9.643667 | 9.433 | 0.210667 | 0.091075 | 2.313113 | 0.472021 |
| T1 | T12 | 9.643667 | 9.578667 | 0.065 | 0.091075 | 0.713698 | 0.9 |
| T1 | T2 | 9.643667 | 9.644333 | -0.00067 | 0.091075 | -0.00732 | 0.9 |
| T1 | T3 | 9.643667 | 9.600333 | 0.043333 | 0.091075 | 0.475798 | 0.9 |
| T1 | T4 | 9.643667 | 9.461 | 0.182667 | 0.091075 | 2.005673 | 0.664518 |
| T1 | T5 | 9.643667 | 9.533333 | 0.110333 | 0.091075 | 1.211456 | 0.9 |
| T1 | T6 | 9.643667 | 9.455667 | 0.188 | 0.091075 | 2.064233 | 0.628087 |
| T1 | T7 | 9.643667 | 9.416333 | 0.227333 | 0.091075 | 2.496112 | 0.346781 |
| T1 | T8 | 9.643667 | 9.417 | 0.226667 | 0.091075 | 2.488792 | 0.351631 |
| T1 | T9 | 9.643667 | 9.322667 | 0.321 | 0.091075 | 3.524569 | 0.023921 |
| T10 | T11 | 9.360667 | 9.433 | -0.07233 | 0.091075 | -0.79422 | 0.9 |
| T10 | T12 | 9.360667 | 9.578667 | -0.218 | 0.091075 | -2.39363 | 0.416938 |
| T10 | T2 | 9.360667 | 9.644333 | -0.28367 | 0.091075 | -3.11465 | 0.083509 |
| T10 | T3 | 9.360667 | 9.600333 | -0.23967 | 0.091075 | -2.63153 | 0.266473 |
| T10 | T4 | 9.360667 | 9.461 | -0.10033 | 0.091075 | -1.10166 | 0.9 |
| T10 | T5 | 9.360667 | 9.533333 | -0.17267 | 0.091075 | -1.89587 | 0.732832 |
| T10 | T6 | 9.360667 | 9.455667 | -0.095 | 0.091075 | -1.0431 | 0.9 |
| T10 | T7 | 9.360667 | 9.416333 | -0.05567 | 0.091075 | -0.61122 | 0.9 |
| T10 | T8 | 9.360667 | 9.417 | -0.05633 | 0.091075 | -0.61854 | 0.9 |
| T10 | T9 | 9.360667 | 9.322667 | 0.038 | 0.091075 | 0.417239 | 0.9 |
| T11 | T12 | 9.433 | 9.578667 | -0.14567 | 0.091075 | -1.59941 | 0.9 |
| T11 | T2 | 9.433 | 9.644333 | -0.21133 | 0.091075 | -2.32043 | 0.467138 |
| T11 | T3 | 9.433 | 9.600333 | -0.16733 | 0.091075 | -1.83731 | 0.769265 |
| T11 | T4 | 9.433 | 9.461 | -0.028 | 0.091075 | -0.30744 | 0.9 |
| T11 | T5 | 9.433 | 9.533333 | -0.10033 | 0.091075 | -1.10166 | 0.9 |
| T11 | T6 | 9.433 | 9.455667 | -0.02267 | 0.091075 | -0.24888 | 0.9 |
| T11 | T7 | 9.433 | 9.416333 | 0.016667 | 0.091075 | 0.182999 | 0.9 |
| T11 | T8 | 9.433 | 9.417 | 0.016 | 0.091075 | 0.175679 | 0.9 |
| T11 | T9 | 9.433 | 9.322667 | 0.110333 | 0.091075 | 1.211456 | 0.9 |
| T12 | T2 | 9.578667 | 9.644333 | -0.06567 | 0.091075 | -0.72102 | 0.9 |
| T12 | T3 | 9.578667 | 9.600333 | -0.02167 | 0.091075 | -0.2379 | 0.9 |
| T12 | T4 | 9.578667 | 9.461 | 0.117667 | 0.091075 | 1.291976 | 0.9 |
| T12 | T5 | 9.578667 | 9.533333 | 0.045333 | 0.091075 | 0.497758 | 0.9 |
| T12 | T6 | 9.578667 | 9.455667 | 0.123 | 0.091075 | 1.350536 | 0.9 |
| T12 | T7 | 9.578667 | 9.416333 | 0.162333 | 0.091075 | 1.782414 | 0.803419 |
| T12 | T8 | 9.578667 | 9.417 | 0.161667 | 0.091075 | 1.775094 | 0.807973 |
| T12 | T9 | 9.578667 | 9.322667 | 0.256 | 0.091075 | 2.810871 | 0.179789 |
| T2 | T3 | 9.644333 | 9.600333 | 0.044 | 0.091075 | 0.483118 | 0.9 |
| T2 | T4 | 9.644333 | 9.461 | 0.183333 | 0.091075 | 2.012993 | 0.659964 |
| T2 | T5 | 9.644333 | 9.533333 | 0.111 | 0.091075 | 1.218776 | 0.9 |
| T2 | T6 | 9.644333 | 9.455667 | 0.188667 | 0.091075 | 2.071553 | 0.623531 |
| T2 | T7 | 9.644333 | 9.416333 | 0.228 | 0.091075 | 2.503432 | 0.341968 |
| T2 | T8 | 9.644333 | 9.417 | 0.227333 | 0.091075 | 2.496112 | 0.346781 |
| T2 | T9 | 9.644333 | 9.322667 | 0.321667 | 0.091075 | 3.531889 | 0.023351 |
| T3 | T4 | 9.600333 | 9.461 | 0.139333 | 0.091075 | 1.529875 | 0.9 |
| T3 | T5 | 9.600333 | 9.533333 | 0.067 | 0.091075 | 0.735658 | 0.9 |
| T3 | T6 | 9.600333 | 9.455667 | 0.144667 | 0.091075 | 1.588435 | 0.9 |
| T3 | T7 | 9.600333 | 9.416333 | 0.184 | 0.091075 | 2.020313 | 0.655411 |
| T3 | T8 | 9.600333 | 9.417 | 0.183333 | 0.091075 | 2.012993 | 0.659964 |
| T3 | T9 | 9.600333 | 9.322667 | 0.277667 | 0.091075 | 3.04877 | 0.099278 |
| T4 | T5 | 9.461 | 9.533333 | -0.07233 | 0.091075 | -0.79422 | 0.9 |
| T4 | T6 | 9.461 | 9.455667 | 0.005333 | 0.091075 | 0.05856 | 0.9 |
| T4 | T7 | 9.461 | 9.416333 | 0.044667 | 0.091075 | 0.490438 | 0.9 |
| T4 | T8 | 9.461 | 9.417 | 0.044 | 0.091075 | 0.483118 | 0.9 |
| T4 | T9 | 9.461 | 9.322667 | 0.138333 | 0.091075 | 1.518895 | 0.9 |
| T5 | T6 | 9.533333 | 9.455667 | 0.077667 | 0.091075 | 0.852777 | 0.9 |
| T5 | T7 | 9.533333 | 9.416333 | 0.117 | 0.091075 | 1.284656 | 0.9 |
| T5 | T8 | 9.533333 | 9.417 | 0.116333 | 0.091075 | 1.277336 | 0.9 |
| T5 | T9 | 9.533333 | 9.322667 | 0.210667 | 0.091075 | 2.313113 | 0.472021 |
| T6 | T7 | 9.455667 | 9.416333 | 0.039333 | 0.091075 | 0.431879 | 0.9 |
| T6 | T8 | 9.455667 | 9.417 | 0.038667 | 0.091075 | 0.424559 | 0.9 |
| T6 | T9 | 9.455667 | 9.322667 | 0.133 | 0.091075 | 1.460335 | 0.9 |
| T7 | T8 | 9.416333 | 9.417 | -0.00067 | 0.091075 | -0.00732 | 0.9 |
| T7 | T9 | 9.416333 | 9.322667 | 0.093667 | 0.091075 | 1.028457 | 0.9 |
| T8 | T9 | 9.417 | 9.322667 | 0.094333 | 0.091075 | 1.035777 | 0.9 |

*Statistical differences exist between T1-T9 and T2-T9 at the p=0.05 level*
